# Treatment of diffuse alveolar hemorrhage secondary to lupus erythematosus with recombinant activated factor VII administered with a jet nebulizer

**DOI:** 10.1186/s40560-014-0047-2

**Published:** 2014-08-27

**Authors:** Raúl Carrillo Esper, Isis Espinoza de los Monteros Estrada, Teresa de la Torre León, Agustín Omar Rosales Gutiérrez, Jorge Arturo Nava López

**Affiliations:** Unidad de Terapia Intensiva, Fundación Clínica Médica Sur, Puente de Piedra 150, Col. Toriello Guerra. Delegación Tlalpan, Mexico, DF 14050 Mexico

**Keywords:** Diffuse alveolar hemorrhage, DAH (recombinant activated factor VII), Systemic lupus erythematosus (SLE)

## Abstract

Diffuse alveolar hemorrhage (DAH) is a serious pulmonary complication in patients with autoimmune diseases who are undergoing chemotherapy or have had hematopoietic stem cell transplantation. The use of recombinant factor VIIa (rFVIIa) to treat the acute phase of DAH by endobronchial bronchoscopy has been shown to have a significant clinical impact on the survival and evolution of these patients. We report a clinical case of a patient with DAH secondary to systemic lupus erythematosus (SLE) who was treated with rFVIIa administered using a jet nebulizer, obtaining an adequate hemostatic effect with immediate control of DAH and a significant improvement in gas exchange.

## Background

Diffuse alveolar hemorrhage (DAH) is a serious pulmonary complication secondary to autoimmune diseases, chemotherapy, and stem cell transplantation. Its immunopathogenesis has been linked to the release of inflammatory cytokines, which are involved in alveolar capillary endothelial injury and inflammation. Interstitial alveolar inflammation influences the expression of the intra-alveolar tissue factor (TF), causing an increase in the markers of thrombin generation in bronchoalveolar lavage (BAL) fluid [1-5].

Pulmonary complications in systemic lupus erythematosus (SLE) may occur in 50%–70% of patients. Their clinical spectrum is characterized by pleuritis, interstitial pneumonitis, thromboembolism (mainly associated with antiphospholipid syndrome), nodules, bronchiolitis obliterans, infections, diaphragmatic weakness, and DAH. DAH is a rare manifestation that occurs in 2% to 5.4% of SLE patient, and it is associated with high mortality, reaching 50% to 80% [6]. DAH can even occur in patients treated with steroids and with good disease control. The treatment of this complication is mainly focused on controlling the immune dysfunction and the inflammatory process, using steroids, cyclophosphamide, and plasmapheresis [6-10].

DAH treatment *per se*, regardless of its diverse etiology, includes the use of recombinant activated factor VII (rFVIIa), administered bronchoscopically or intravenously, but no information is yet available regarding the use of this medication inhaled using a jet micronebulizer. The aim of this paper is to report the case of a female patient with SLE and DAH who was treated during the active phase of bleeding with inhaled rFVIIa using a jet micronebulizer.

## Case presentation

A 37-year-old female patient with a history of SLE and Sjögren syndrome was treated with deflazacort 30 mg/day. The patient was admitted to the hospital with a clinical picture characterized by abdominal pain, chills, and fever. Abdominal computed tomography (CT) showed diverticulitis Hinchey III and free fluid in the pelvic cavity, and the patient underwent laparoscopic surgery, confirming the CT findings and detecting a sealed diverticular perforation.

**Figure 1:**
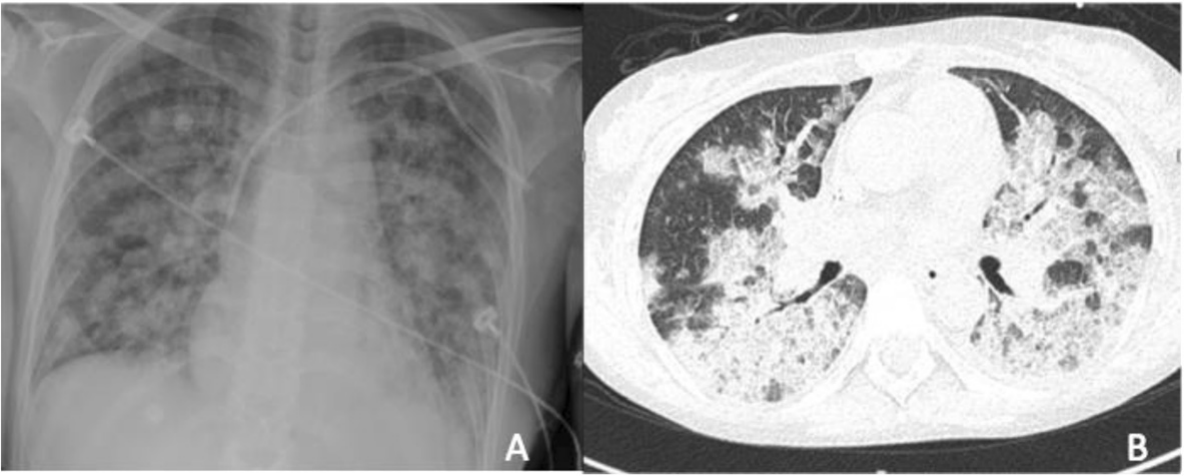
**Chest X-ray and CT scan. (A)** Chest X-ray where bilateral pulmonary infiltrates were observed. **(B)** Computed axial tomography (CT) scan where mixed basal pulmonary infiltrates and alveolar collapse were observed. Images were taken at the time of admission (day 1).

Sudden tachycardia, tachypnea, hypoxemia, cough, and hemoptysis appeared in the immediate postoperative period, and the patient was transferred to the intensive care unit (ICU) with respiratory failure and generalized coarse rales on admission. The cough increased and the hemoptysis became more evident, reaching approximately 200 mL. The patient had hypoxemia, with 86% oxygen saturation with 100% FiO_2_ by pulse oximetry. The blood gas analysis on admission was as follows: pH 7.18, pCO_2_ 39 mmHg, pO_2_ 42.3 mmHg, lactate 3.3 mmol/L, alveolar-arterial gradient 455 mmHg, and short circuits 50%. Hemoglobin decreased from 10 to 7 g/dL. Chest radiographs showed bilateral pulmonary infiltrates occupying all four quadrants, confirmed by chest CT, on the day of admission (Figure 1).

Echocardiography showed a dilated right atrium and ventricle, tricuspid regurgitation, and pulmonary artery systolic pressure of 42 mmHg. A rheumatic profile was performed with hypocomplementemia results for C3 of 21.2 mg/dL (70–152 mg/dL) and C4 of 4.2 mg/dL (16–38 mg/dL), positive speckled pattern antinuclear antibodies (ANA) at a titer of 1:1,640, and granular cytoplasmic fluorescence. Anticentromere antibodies (antineutrophil cytoplasmic antibody (ANCA)) were c-ANCA <1:20 (neg <1:20), p-ANCA <1:20 (neg <1:20), and atypical p-ANCA <1:20 (neg <1:20); erythrocyte sedimentation rate (ESR) was 60 mm/h (0–7.4 mm/h); and C-reactive protein (CRP) was 71 mg/L (0–20 mg/L).

After the diagnosis of DAH secondary to SLE, treatment was started with rFVIIa at a dose of 50 μg/kg inhaled with a jet nebulizer, achieving rapid DAH control, with the disappearance of hemoptysis and a rapid increase in PaO_2_ and the PaO_2_/FiO_2_ ratio.

**Figure 2:**
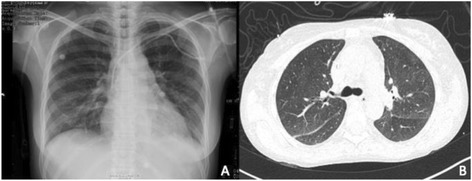
**Chest radiography and CT scan. (A)** Radiography of the chest after treatment with rFVIIa, steroids, and rituximab where the resolution of pulmonary infiltrates was observed. **(B)** Chest CT scan where the resolution of infiltrates and alveolar collapse was confirmed (7th day).

Three boluses of 1 g methylprednisolone were administered, and 500 mg rituximab was later added to therapy. The patient's condition improved, with follow-up radiographs and a CT scan showing the resolution of the infiltrates, after 24 h (Figure 2).

## Discussion

DAH is a rare complication of SLE with high mortality. The most common age of onset is around 30 years [11,12]. DAH usually occurs in patients with an established diagnosis of SLE; however, it can also be the initial manifestation of this disease [13,14].

The proposed mechanisms for DAH development include precapillary and alveolar space damage mediated by immune processes. Three different patterns have been described histopathologically: subclinical hemorrhage, capillaritis, and diffuse alveolar damage [15-18].

Most cases are associated with slight bleeding without any sign of vasculitis, interstitial inflammation, or infection; however, scientific evidence has shown an increase in the incidence of capillaritis [19,20].

Patients with SLE who present a triad of hemoptysis, sudden drop in hemoglobin in 24–48 h, and new alveolar or interstitial infiltrates should be highly suspected of DAH. The incidence of hemoptysis varies, and it is not present clinically in some cases, confirmed by bronchoscopy and bronchoalveolar lavage fluid examination that shows erythrocytes and hemosiderin-laden macrophages. DAH is associated with other symptoms and signs such as fever, dyspnea, cough, and pleural effusion. The most common extrapulmonary manifestation of SLE is renal involvement, which usually manifests as nephritis class III or IV. A higher risk of DAH has been found in patients with active renal disease, especially nephrotic syndrome. Serological analysis in these patients shows a distinctive high anti-dsDNA titer, hypocomplementemia, and anemia, as was the case in our patient. With regard to the radiographic findings, diffuse alveolar infiltrates were evident from plain radiographs and CT, but did not involve the lung apices [6,12,19-23].

Bronchoscopy has been recommended in the case of suspicion of DAH in which clinical presentation is atypical, as it confirms the diagnosis. In subacute cases, methods such as the quantitative score of hemosiderin-laden macrophages in the BAL fluid sample or increased red blood cell counts are used [6]. Hemosiderin, a breakdown product of hemoglobin, is not present in all cases in the acute and early stages, as it appears at least 48 h after bleeding [24,25].

The treatment of DAH is based on high doses of steroids. This therapeutic approach achieves good results, especially when administered early. Cyclophosphamide or plasmapheresis can be added to the treatment when the response is not satisfactory [26,27].

Mendoza et al. [28] reviewed the treatment of patients with DAH secondary to SLE, exploring the therapeutic potential of rituximab (anti-CD20 monoclonal antibody). Most cases showed significant improvements in clinical and laboratory parameters, with good tolerance and few side effects. Thus, patients with severe lupus nephritis showed improvement in the disease activity with a significant reduction (*p* < 0.05) of proteinuria (from 3.710 to 1.786 g/L, *p* < 0.05). Patients with severe neurological involvement had complete symptom remission.

The rFVIIa is a medication approved for the treatment of hemorrhage in patients with hemophilia A or B who produce antibodies to FVIII or IX. FVII initiates clot formation by interaction with TF. The FVIIa-TF complex activates factor X. At high doses (80 to 100 mg/kg), activated FVII also activates factor X in the absence of TF, most likely via the activation of factor X bound to the surface of activated platelets. Factor Xa activates prothrombin to thrombin, which in turn converts fibrinogen to fibrin [29,30].

It has been proven that rFVIIa has a hemostatic effect in DAH patients of different etiologies [31-33]. Heslet et al. [34] published one case series in which they evaluated the use of rFVIIa in six patients with DAH, in which rFVIIa was instilled into the bronchial system by bronchoscopy at a dose of 50 μg/kg with good results, so it was decided to use this dose in our patient. Carrillo et al. [35] reported the case of a patient with DAH secondary to SLE who responded well to intravenous rFVIIa. Grochova et al. [36] reported a case of acute DAH secondary to stem cell transplantation, confirmed by bronchoscopy, which successfully responded to the administration of rFVIIa at a dose of 50 μg/kg in each of the intrapulmonary main bronchi by bronchoscopy. Dabar et al. [37] reported two DAH cases secondary to acute leukemia and granulomatous vasculitis, respectively, both successfully treated with intrabronchial rFVIIa administered by bronchoscopy. Estella et al. [38] reported two cases of DAH secondary to SLE, both successfully treated with the intrabronchial instillation of 90 μg/kg rVIIa by bronchoscopy. rFVIIa is a glycoprotein of 406 amino acids, has a synthetic G_0_ plasma activity analogous to that of FVII, has a molecular weight of 50 kDa, and has a half-life of 3 h. Its effect is immediate and clinical hemostasis is observed in 10 min. It was developed in the 1980s for the treatment of bleeding in patients with congenital hemophilia with inhibitors and in some cases for patients with congenital FVII deficiency. Since the 1990s, the first series published about its use were in non-hemophiliac patients with severe hemorrhage of different etiologies [38,39].

The dose of rFVIIa used in different reports is 40–300 μg/kg. The recommended dose in patients with microvascular bleeding is 60 to 100 μg/kg bolus, with a second dose after 20 min if they had not obtained satisfactory hemostatic control. In our work, we administered a first dose of 100 μg/kg, and due to rebleeding, a second dose was necessary to obtain appropriate response [40].

In all published cases related to the use of rFVIIa in DAH, this medication has been administered by an intrabronchial or intravenous route. In the case of our patient, the route of administration was by inhalation using a jet micronebulizer, obtaining a favorable outcome, which opens a new opportunity and route of administration for the use of rFVIIa in DAH. This new approach is supported on the basis that hemostasis can be achieved by the interaction of rFVIIa and TF expressed at the alveolar level when an immune/inflammatory process is taking place [41].

The mechanism of action of alveolar hemostasis is primarily explained by the TF-dependent pathway, where alveolar TF is expressed during the inflammatory phase of DAH. Moreover, the tissue factor pathway inhibitor (TFPI) inhibits the local activation of factor X to Xa by the FVIIa-TF complex. In acute lung injury, the TFPI produced by alveolar macrophages can increase up to 20 times. Our observations indicate that the intrapulmonary administration of FVIIa negates TFPI's anticoagulant effect [42-44].

Clinical trials within the approved indications revealed that thrombotic events of possible or probable relationship to rFVII occurred in 0.28% of bleeding episodes treated, with the incidence within hemophilia patients with inhibitors to be 0.20%, and in acquired hemophilia an incidence of 4% [45]. Patients with disseminated intravascular coagulation (DIC), advanced atherosclerotic disease, crush injury, septicemia, or concomitant treatment with activated or nonactivated prothrombin complex concentrates (aPCCs/PCCs) have an increased risk of developing thrombotic events.

Two meta-analyses of these pooled data indicate an increased risk of thrombotic events (10.0% in patients treated with rFVIIa versus 7.5% in placebo-treated patients). Arterial thromboembolic adverse events including myocardial infarction, myocardial ischemia, cerebral infarction, and cerebral ischemia were statistically significantly increased with the use of rFVIIa compared to placebo (5.3% to 5.6% in subjects treated with rFVIIa versus 2.8% to 3.0% in placebo-treated patients). Other arterial thromboembolic events (such as retinal artery embolism, renal artery thrombosis, arterial thrombosis of limb, bowel infarction, and intestinal infarction) have also been reported [45-49].

## Conclusion

The DAH in SLE is a catastrophic complication and is associated with high morbidity and mortality. The use of rFVIIa by inhalation with a jet nebulizer is an option for the treatment of this condition, and it should always be given as a supplement to the standard treatment (methylprednisolone boluses).

## Consent

The patient has given consent for the publication of accompanying documents. The authors are available for any clarification. The study was approved by the ethics committee of the Hospital Medica Sur which is governed by the Declaration of Helsinki.

## Abbreviations

ANA: antinuclear antibodies
ANCA: anticentromere antibodies
BAL: bronchoalveolar lavage
CRP: C-reactive protein
CT: computed tomography
DAH: diffuse alveolar hemorrhage
ICU: intensive care unit
rFVIIa: recombinant activated factor VII
SLE: systemic lupus erythematosus
TF: tissue factor
TFPI: tissue factor pathway inhibitor
